# Extended mathematical cognition: external representations with non-derived content

**DOI:** 10.1007/s11229-019-02097-w

**Published:** 2019-01-28

**Authors:** Karina Vold, Dirk Schlimm

**Affiliations:** 1 Leverhulme Centre for the Future of Intelligence, Faculty of Philosophy, Cambridge University, Level One, 16 Mill Lane, Cambridge, UK; 2 Department of Philosophy, McGill University, 855 Sherbrooke St. West, Montreal, QC Canada

**Keywords:** Mental content, Representational vehicles, Vehicle externalism, Cognitive integration, Enculturation, Mathematical cognition

## Abstract

Vehicle externalism maintains that the vehicles of our mental representations can be located outside of the head, that is, they need not be instantiated by neurons located inside the brain of the cogniser. But some disagree, insisting that ‘non-derived’, or ‘original’, content is the mark of the cognitive and that only biologically instantiated representational vehicles can have non-derived content, while the contents of all extra-neural representational vehicles are derived and thus lie outside the scope of the cognitive. In this paper we develop one aspect of Menary’s vehicle externalist theory of cognitive integration—the process of enculturation—to respond to this longstanding objection. We offer examples of how expert mathematicians introduce new symbols to represent new mathematical possibilities that are not yet understood, and we argue that these new symbols have genuine non-derived content, that is, content that is not dependent on an act of interpretation by a cognitive agent and that does not derive from conventional associations, as many linguistic representations do.

## Introduction

Philosophers of mind have traditionally believed that our representations are instantiated by states of the brain and, thus, located ‘internally’, or entirely in one’s head. But more recently, vehicle externalists have challenged this tradition, instead maintaining that representational vehicles can be located outside of the head. Although there are now different strands of vehicle externalism (e.g., Menary 2007, 2015; Clark and Chalmers 1998), most argue that environmental states and processes can be seamlessly integrated with the functions of our neural states and processes, making the two—external and internal states and processes—equally essential for some aspects our cognitive life. On this view, cognitive processes are not merely ‘scaffolded’ by tools and structures in the environment (Vygotsky 1930)—they are partially *constituted by*, rather than merely *causally dependent* on, external structures.

One common objection confronting vehicle externalism maintains that non-derived content is the mark of the cognitive but no external representations could have non-derived content—thus, no external representations could be genuinely cognitive (Adams and Aizawa 2001, 2008, 2010; Aizawa and Adams 2005). In this paper, we offer a new response to this longstanding objection by focusing on cases of mathematical cognition. Our strategy will be to grant non-derived content as a mark of the cognitive but demonstrate that external representations—in particular, certain mathematical symbols—can have non-derived content and should therefore count as genuinely cognitive. Mathematicians sometimes rely on external markers as vehicles, which they manipulate in computations in order to understand their content—a process known as *operative writing* (Krämer 2003). Developing and understanding the content of these symbols can be a long process involving a community of thinkers. We argue that the contents of these symbols are not derived from any other representational state, nor are they derived from purely conventional associations. Rather, they stand as an example of external, non-biologically instantiated representations with non-derived content.

## Vehicle internalism and externalism

Traditional cognitive science subscribes to the view that whatever else is true of cognitive processes, they take place entirely in the brain, such that (under the right conditions) the operations of the brain could function independently of the wider body. According to this view, known as *internalism*, mental representations, and the relevant computations over these representations, are instantiated by states and processes of the brain. As a consequence, this view allows for the possibility that a brain floating in a vat could enjoy the same mental life as an embodied brain, despite being removed from a biological body (Fodor 1980, 1981). Hence, while rejecting substance dualism as a metaphysical position, traditional cognitive science still holds on to the Cartesian idea that the mind is, in some sense, distinct from the body and the world it inhabits, in the sense that it can be disassociated or separated from these external factors, at least for the purpose of explanation.

A key distinction amongst varying internalist positions on mental representations hinges on the difference between the *vehicles* and the *contents*. The vehicle of a representation is the physical structure that represents, while the content is that which is represented. Accordingly, *vehicle internalism* (also known as *intracranialism*) is the view that all of the vehicles of our mental representations are within the skull, while *content internalism* argues that the contents of our mental representations are fixed, or determined, by properties of our brains. Burge (1979) famously argues against content internalism, developing Putnam’s (1975) twin-earth arguments against semantic internalism. Vehicle internalism, on the other hand, has only met popular challenges and rival views more recently, including Menary’s (2007) theory of cognitive integration and Clark and Chalmers’s (1998) extended mind thesis, among other views.^1^ The central argument in the present paper defends vehicle externalism from the common objection that external representations could not have cognitive content. To this end, it is worth noting that the vehicle internalist/externalist distinction is often thought to vary independently of the content internalist/externalist positions (Chalmers 2008; Theiner 2011). As we will see, however, the popular objection confronting vehicle externalism that we focus on has to do with the nature of the content that is represented by the vehicles of mental representations. As a result, the relations between vehicles and their contents become important for our argument.

## Vehicle externalism: the extended mind thesis, cognitive integration, and enculturation

There are different versions of vehicle externalism, and for the sake of brevity we focus on two in particular. The first is Clark and Chalmers’s extended mind thesis. Given that it was this thesis against which the objection we respond to in this paper—the objection from non-derived content—was originally lodged, an introduction of this view will set the stage for the present discussion. But, as will see, this objection applies to all versions of vehicle externalism. The second view of vehicle externalism we discuss is Menary’s theory of cognitive integration. Menary’s theory helps to provide a way of responding to the objection from non-derived content.

### Clark and Chalmers’s extended mind thesis

Clark and Chalmers’s extended mind thesis maintains that our mental states and cognitive processes can be instantiated by physical states outside our brain and body.^2^ Defenders of this thesis accept the computational theory of mind but argue that when we use tools, such as a pen and paper or a calculator, they can become seamlessly integrated into our cognitive processes, such that computations (that is, the manipulations of representations in accordance with formal rules) in the tools are just as essential to our cognition as the computations in our brain. In this sense, the tool ‘extends’ our cognition. Put more precisely, the information-bearing structures within the tool are the vehicles of genuine mental representations, as are the vehicles of neurally instantiated mental representations. Vehicle internalism (henceforth ‘*intracranialism*’) is therefore rejected. To motivate support for their view, Clark and Chalmers (1998) offer the following principle:

If, as we confront some task, a part of the world functions as a process which, *were it done in the head*, we would have no hesitation in recognizing as part of the cognitive process, then that part of the world *is* (so we claim) part of the cognitive process. (p. 8)

Many now refer to this as Clark and Chalmers’s ‘parity principle,’ while others call it the ‘fair treatment principle’ (e.g., Sprevak 2009; Drayson 2010), as it maintains that we should treat equivalent processes with “the parity they deserve” irrespective of whether they are internal or external to the skull (Clark and Chalmers 1998, p. 8). Armed with their parity principle, Clark and Chalmers describe a case in which, they argue, an object in the environment plays the same role for one agent that neurons in the brain (something we would surely count as a part of the supervenience base of the mind) do for another. The case involves two people, Inga and Otto. Inga decides to go to an exhibition at the museum; to do so, “[s]he thinks for a moment and recalls that the museum is on 53rd Street, so she walks to 53rd Street and goes into the museum” (Clark and Chalmers 1998, p. 12). Meanwhile, we imagine that Otto suffers from Alzheimer’s disease and has to rely on information he stores in a notebook to help structure his life. When he decides to go to the same exhibition, he consults his notebook, where he has written the address and directions to get there. He then walks to the museum and goes inside. Clark and Chalmers maintain that in the relevant respects, the symbols in Otto’s notebook (i.e., the words on the page) function just as the internal representational vehicles in Inga’s brain that constitute an ordinary belief, and so both should count equally as part of the constitutive machinery of their beliefs. Further, since beliefs are widely considered a part of one’s mind, it follows that a part of Otto’s mind is constituted by a resource located outside of his brain. It is in this sense that Clark and Chalmers maintain that our minds can extend beyond our biological bodies.

### Menary’s theory of cognitive integration

Menary’s theory of cognitive integration shares the commitment to vehicle externalism and the rejection of intracranialism, but it has a different aim and scope from the extended mind thesis. Cognitive integration explains how our minds become enculturated, where *enculturation* refers to the transformation of biological faculties through the period of cognitive development, learning-driven neural plasticity, and cultural environments (Menary 2015). The ‘cultural environment’ includes our cognitive practices, mathematical and linguistic symbols, and complicated social worlds—what philosophers now refer to as our ‘cognitive niche.’^3^ Cognitive practices include the cultural formalization of patterns of action across populations and groups, and these are, crucially, brought about through the creation and manipulation of representational vehicles (or information-bearing structures) in public spaces, that is, ‘external’ spaces (Menary 2007, p. 4). Menary (2015) uses the case of mathematical cognition as a primary example of enculturation. We will focus on two concepts that characterize the process of enculturation that are most important for our later discussion of mathematical cognition: evolutionary continuity and transformation.^4^

Evolutionary continuity refers to the gradual progress of evolution that takes place through complex structures, including cultural, social, biological, neural, and genetic structures, among others, over many generations. Much research has been done on the evolutionary continuity of human brains from our archaic predecessors and even lower primates.^5^ But enculturation focuses on another sense of evolutionary continuity, which is key to understanding current mathematical practices, namely, the continuity between biology and culture. Humans are born into highly structured mathematical cultures that contain representational systems, such as numeral systems, that embody knowledge, as well as practices and methods for manipulating these representations (Ferreirós 2015; Larvor 2016). A budding mathematician does not invent a new number system; she learns the skills and practices of her cognitive niche and then learns methods for attaining new knowledge (Menary and Kirchhoff 2014). Menary (2015) sees these cultural practices as intertwined with our biological capacities: together they allow for mathematical cognition. He refers to this kind of evolutionary continuity as ‘cultural evolution’. Crucially, cognitive practices are the products of cultural evolution, which can evolve over much faster timescales than biology. Thus, cultural evolution can allow for new cognitive practices to emerge without being slowed down by biological adaptations. Menary’s appeal to cultural evolution indicates how his theory of cognitive integration offers a multi-layered approach to understanding cognition—one that considers the unique contributions of the body, environment, culture, and evolution, in addition to the brain.^6^ In doing so it also commits to the claim that the representational vehicles of an organism’s cognitive states and processes are fundamentally integrated with environmental states and processes, such that the ‘inner’ and ‘outer’ form a single, unified cognitive system.

A second concept that characterizes the process of enculturation is the ‘transformation thesis’. As Menary (2015) articulates the thesis: ‘[C]ognitive transformations occur when the development of the cognitive capacities of an individual are sculpted by the cultural and social niche of that individual’ (p. 8). The manipulations of mathematical symbols, such as numerals and operators, are governed by culturally established cognitive norms, which mathematicians must learn in order to engage in correct mathematical practices. It is through learned norms about how to correctly manipulate inscriptions that mathematicians are able to grasp the content of newly introduced mathematical symbols. For Menary, this demonstrates a transformation of our cognitive—not biological—capacities. Our cognitive capacities are also transformed by the use of mathematical symbols and axioms. These external structures are not just needed for scaffolding during the learning process and then removed after the new cognitive capacities are in place. Rather, mathematical symbols and practices are permanent devices—or extensions—that even the most expert mathematicians rely on in their cognitive practices, even if these are internalized (Schlimm 2018). These symbols and axioms thus transform how mathematical cognition occurs.^7^

Insofar as cognitive integration and the extended mind thesis both commit to vehicle externalism, they face a similar set of objections. Perhaps the most significant objection is that our cognition is merely causally dependent upon, rather than partially constituted by, external states and processes. As we will explain, this line of objection risks a stalemate unless one side can provide a principled reason (i.e., one that does not beg the question) for preferring the causal claim to the constitutive claim, or vice versa. It is this debate that has led proponents of intracranialism to advocate for non-derived content as a ‘mark of the cognitive’, as a way of deciding how to draw a non-question-begging line between the mind (for them, the brain) and the world. We will argue against this proposed mark of the cognitive by developing an aspect of Menary’s ideas on mathematical cognition as a response to this objection.

## Alternative views: is the mind extended or embedded?

The distinction between the mere causal dependence of inner and outer resources and the constitutive involvement of outer resources is what distinguishes the *embedded* theory of cognition from the stronger *vehicle externalism* view.^8^ The embedded account maintains that a cognitive system may crucially depend on the complexity of its environment but that the environment is not an actual part of the mind.^9^ Simon (1969), for example, argues that much of the apparent complexity of cognitive systems is actually external to the agent and residing in the environment. On this view, cognitive systems lean heavily on worldly complexity without internalizing it. For example, humans sometimes structure their own environment to store information and then rely on these external structures instead of on internal resources. The embedded account would explain Otto’s interaction with his notebook in these terms: Otto writes into his notebook, thereby structuring his environment, and then relies on these external information-bearing structures to get around the world instead of relying on his internal resources. For Otto, this might be necessary because his internal information-bearing structures (i.e. neurons in his brain) are no longer reliable—but we could imagine that Inga, who does not suffer from Alzheimer’s, also decides to rely on a notebook instead of her own well-functioning internal resources. The *mise en place* method of lining up one’s ingredients in the correct order for cooking, for instance, is often used by chefs to spare them the task of remembering the ordering of their recipes while cooking (Kirsh 1995; Clark 2008). The embedded view of cognition tells us that in order to understand and explain cognitive processes, such as the chef’s use and processing of information while cooking, cognitive science cannot just study the internal processes of computation instantiated in the brain. Instead, we must study the way that structures in the local environment of an agent *facilitate* the success of the agent’s internal processes. Thus, the embedded view offers an explanatory, or epistemic, reason to look beyond the brain, but it does not make any substantial constitutive claim—it does not challenge the metaphysical view that the brain wholly constitutes the mind (Rowlands 2010). The embedded view would say that all of Otto and Inga’s beliefs are in their head, and Otto thus lacks any belief about where the museum is located. If Inga cannot recall where the museum is located and has to retrieve this information from her own notebook, then she too would lack a belief about where the museum is located, whereas if she stored the information internally, then she would have that belief. The key difference then is that embedded mind theorists accept intracranialism, while extended mind theorists reject it.

Vehicle externalists tend to think that the embedded view risks triviality since almost everyone agrees that the mind is in some sense causally reliant on both the body and the extra-bodily world. Embedded mind theorists, on the other hand, argue that there is no good reason for preferring the stronger constitutive claim to the more conservative causal-dependence claim.^10^ Thus, a stalemate looms unless we can find a way to arbitrate between these two views. Clark and Chalmers (1998) maintain that the burden of proof lies with those who reject the constitutive claim—they must offer a principled reason for insisting that all mental states are entirely constituted by neural resources and only causally supported by extra-neural ones. Simply pointing to the skull as the relevant boundary would amount to begging the question. To this end, Adams and Aizawa (2001) argue that non-derived content is the distinguishing ‘mark of the cognitive’.

## Objection from non-derived content

In response to Clark and Chalmers’s argument for vehicle externalism, Adams and Aizawa (2001, 2008, 2010) counter that there is a ‘mark of the cognitive’ that distinguishes representations that are genuinely *cognitive* from those that are not. A necessary condition for a state or process to be cognitive, they argue, is for it to bear *non*-*derived* content—sometimes called ‘original’ or ‘intrinsic’ content.^11^ Thus, all cognitive states represent intrinsically, while non-cognitive representational states *derive* their meanings from conventions, social practices, or the representational states of cognitive agents, which are themselves not derived from anything.^12^ Exactly how neural states come to have non-derived content has been one of the major topics for debate in philosophy of cognitive science over the last few decades. Adams and Aizawa (2001, p. 48) acknowledge that there is no consensus, nor do they take a position on this issue, but they maintain there is ‘a fairly broad consensus that cognition involves non-derived content’. They further maintain that, as a matter of contingent fact, our biologically instantiated mental representations are the only things with intrinsic content. They thus label themselves ‘contingent intracranialists’: while vehicle externalism is logically possible, they maintain that the world happens to be such that there are no actual cases of it.

To illustrate the notion of intrinsic content, let us return to the case of Otto and Inga. The words in Otto’s notebook derive their content either through conventional associations or from his own internal and biologically instantiated representational capacities,^13^ such as his (internal) cognitive states, whereas Inga’s belief does not represent the location of the museum by virtue of any convention or social practices. Her neural states carry this content in some other, more direct way (again, exactly how neural states carry meaning is an issue of controversy). Like the symbols in Otto’s notebook, all other external representations lack intrinsic content, at least according to Adams and Aizawa. Thus, the non-derived content condition provides a principled reason for preferring intracranialism and the causal-dependence claim of the embedded mind thesis, to the constitutive claim made by the extended mind thesis. Cognitive processes may be causally supported by non-neural states and processes, but all and only cognitive states and processes have non-derived content.^14^ This is the objection from non-derived content that Adams and Aizawa use to defend contingent intracranialism.^15^ Thus, if we grant Adams and Aizawa their proposed mark of the cognitive, the challenge confronting vehicle externalist theories is to show not just the modal claim that external representations *could* have non-derived content, but that they in fact do, at least in some cases. If this can be shown, then contingent intracranialism would be proven false.

## Responses to the objection from non-derived content

Many have offered various replies to counter the objection from non-derived content. First, some express scepticism that the distinction between derived and non-derived content is even coherent (Dennett 1990, 1986; Prinz and Clark 2004; Clark 2005, 2010). Second, assuming the distinction is coherent, it would be question-begging to assume a priori that only internal biological representations can have non-derived content without some further reason explaining why this is (Prinz and Clark 2004; Clark 2010). Third, even if one accepts the distinction between non-derived and derived content *and* one accepts that external representations are incapable of non-derived content, one could deny that non-derived content really is the mark of the cognitive on the grounds that there could be genuinely cognitive, *internal* states whose content is derived (see Clark 2005, 2010; Wheeler 2017). A fourth response is to grant non-derived content as a mark of the cognitive but to argue that internal subprocesses with non-derived content can work in conjunction with external subprocesses such that the overarching external or extended cognitive process will involve computations over representations with non-derived content, even if some of the content is derived (e.g., Clark 2008; Wheeler 2010a, b, 2014, 2017).

Notably, all of these strategies admit that the relevant external representations lack non-derived content. The objective of this paper is to offer a new response to this longstanding challenge. Our strategy will be to grant non-derived content as a mark of the cognitive but argue that (at least some) external representations can have non-derived content and, thus, can count as genuinely cognitive. One example of external representations with non-derived content are cases of *social extension,* where the mental states of one individual are partially constituted by the mind of another agent. For example, instead of relying on the representational states in his notebook, Otto might rely on the representational states in Inga’s head, such as by asking her for directions to the museum. This would require that Inga play a big part in Otto’s daily life, so that the information in her head plays a similar functional role in Otto’s memory as it does in her own. In this case, even though the representational vehicles are extracranial—external to Otto’s brain—they would still have non-derived content. But in this case the representational states in Inga’s head are still biologically instantiated and thus, according to Adams and Aizawa’s view, should have non-derived content. Our argument will challenge this neuro-chauvinistic commitment by arguing that non-biologically instantiated external symbols can also have non-derived content. Our focus will be on what Menary describes as a primary example of enculturation—mathematical cognition.

First, it is helpful to recall the distinction made earlier between content internalism/externalism and vehicle internalism/externalism as this distinction is now useful to the present debate. Notice that Adams and Aizawa appeal to a particular kind of representational content—non-derived content—to reject vehicle externalism. But, as mentioned earlier, it is widely agreed that the vehicle internalist/externalist distinction varies independently of the content internalist/externalist positions (Chalmers 2008; Theiner 2011). Making clear how this is possible is the first step in our response to the objection from non-derived content.

## Extended internalism and active content externalism

Adams and Aizawa maintain that representations can have content that is either derived or non-derived, and that (as a matter of contingent fact) only neurally instantiated representational vehicles are capable of the latter. They then argue that because non-derived content is a necessary condition for cognition, all non-neurally instantiated representational vehicles are necessarily non-cognitive, and, thus, vehicle externalism is false. We will argue that there can be cases of external vehicles with non-derived content. To do this, we first need to make clear how content internalism/externalism and vehicle internalism/externalism can be separated. Recall that content internalists maintain that representational content is determined by properties of the individual. But contingent intracranialists, like Adams and Aizawa, could endorse content externalism, the view that content is determined by more than just the individual. Burge’s (1979) social externalism, for example, holds that the content of one’s mental states can supervene on one’s wider linguistic community; for instance, some mental content is determined by the relevant experts in our linguistic communities. Thus, vehicle internalists could be either internalists or externalists about content. The same is true for vehicle externalists. A vehicle externalist could maintain that the content of mental representations is always determined by the individual, including the individual’s extended cognitive states and processes—for Otto this would mean both his brain and his notebook.^16^ Or, a vehicle externalist could be a content externalist, maintaining that the content of one’s mental states can supervene on more than just one’s cognitive system, wherever that might be. For example, when Otto relies on representations of the word *arthritis* in his notebook, this symbol serves as the vehicle for his thoughts about arthritis (it might appear in a sentence he wrote about how arthritis is inflammation in the joints), but the content of this vehicle is determined by the relevant experts in his linguistic community. These examples illustrate the common thought that the vehicle internalist/externalist distinction varies independently of the content internalist/externalist positions (Chalmers 2008; Theiner 2011).

Now, we can begin our response to Adams and Aizawa’s objection by considering a further distinction between two kinds of externalism. In addition to describing their extended mind thesis as a version of vehicle externalism, Clark and Chalmers also label their view ‘active externalism’. In active externalism the relevant external representational vehicles play an *active* role in driving the cognitive process. They contrast this with Burge’s version of content externalism, which they describe as ‘passive’ because natural language communities are very large and removed from the cognitive process of the individual, such that the individual cannot change the overall usage of the words she uses—even those that occur in thoughts that she has. Recently, Lyre (2016) has defended the possibility of ‘active content externalism’, where the individual plays an active role in determining the representational content.^17^ Lyre asks us to imagine the case of a very small linguistic community, composed of just a few speakers, where these speakers are the relevant experts themselves. Using a thought experiment rather than a real case to support this possibility, Lyre (2016) asks us to ‘[t]hink of a gang of youth with their own slang’ (p. 29). In this ‘gang-slang’ case the linguistic community that determines the content of the linguistic symbols is not distant or removed from the relevant representational vehicles whose content is being determined. Instead, the members of the group play an *active* role in determining the content of the slang they use. For example, let us consider gang member G, who is an active member of the small linguistic community. G plays a role in determining the content of the external linguistic symbols that she uses. Let’s imagine further that G graffities a few buildings with an abstract tag symbol, and subsequently G and the other members of her group begin to rely on the presence of the tag to represent which parts of the neighbourhood are safe to them. Over time, G and her group come to depend on these symbols regularly to navigate through their town. Thus, the tag serves as an external vehicle of G’s cognition, yet the content of this tag was determined in part by G, along with other members of her small linguistic community.

This case is still fictional and the details have been idealized for the purpose of illustrating the possibility of active content externalism. But notice that neither G nor any other member of her group determines the content of the tag by themselves. When G first draws the tag, its content is not derived from her internal biologically instantiated mental representations. The vehicle has content, but the community only apprehends the content via their interaction with the symbol—through this they come to learn that it represents a safe place for them. The tag, an external representational vehicle, therefore, has content that is not derived from any preceding biological representations. A case like this has the potential to demonstrate how external vehicles can have non-derived content. Now, as a real case, we will consider expert mathematicians who introduce new mathematical symbols and axioms on paper and who, together with a few other mathematicians, determine the content of the new symbols and axioms through their external manipulations. The cases we appeal to also emphasize the social and cultural practices of mathematicians and, thus, demonstrate how new symbols and axioms can be the product of a process of enculturation.

## External representations with non-derived content

To make our case for external representations with non-derived content, we will first defend content externalism with respect to mathematical knowledge, and then defend vehicle externalism for the case of (at least some) mathematical cognitive processes. We then turn to the possibility of active content externalism and describe cases where the content of mathematical symbols is not derived from any internal biologically instantiated representations.

### Mathematical content externalism and vehicle externalism

For the cognitive integrationist, mathematical practices and concepts are not innate: they are cultural practices and culturally evolved symbols and systems that are learned and deployed to complete mathematical cognitive tasks. At a basic level, we all learn these practices and concepts. But at a certain, higher level of expertise, the community of mathematicians becomes quite small. These thinkers determine the content of the symbols that the rest of us rely on. Ferreirós (2015) has recently developed an account of mathematical practices according to which everyday practices and skills ground the more abstract, genuinely mathematical practices. The latter are characterized by the use of symbolic frameworks for the treatment of abstract subjects and the pursuit of theoretical, as opposed to practical, goals. Accordingly, in terms of Burge’s social externalism, if expert mathematicians introduce a new property of the number zero, for instance, that 5^0^ = 1 or 0! = 1 (De Cruz and de Smedt 2013, p. 6), this changes the truth conditions of our beliefs about zero because the content of our representation of the number zero is determined by the experts of the relevant community. This establishes content externalism in the case of mathematical knowledge.

But many have argued that mathematical cognition also provides a strong case for vehicle externalism. Cognitive integration predicts that spatial properties of the representations—for instance, the arrangement of numerals—of the external symbols we manipulate can affect our mathematical cognition (Menary 2015, p. 14). Bear in mind that external mathematical symbols and their manipulations (in accordance with some system of rules) are permanent structures even for mature mathematicians—they are not impermanent scaffolding used only during learning or developmental periods. Noting this, consider a study by Landy and Goldstone (2007), which found that altering the layout of algebraic formulas could cause undergraduate students to make increased errors. By adding more space between terms that are to be added than between those that are to be multiplied, subjects were misled about the order in which the operations had to be applied and thus were more likely to make mistakes. These results support the cognitive integration framework, as well as vehicle externalism more broadly. They certainly show that the spatial layout of symbols affects our mathematical competence, but they have also been taken as evidence that (some cases of) mathematical cognition are partially constituted by our manipulation of external symbols (Dutilh Novaes 2013; Menary 2015). Additional support for the effect of external representations on mathematical cognition includes the representational effects in mental arithmetic. For example, work by Nuerk et al. (2001) shows that people consider the units and decades separately when determining which of two numbers is greater. This explains the surprising phenomenon that 42 < 53 is generally assessed faster and more accurately than 42 < 61, despite the fact that the numerical distance between the latter two numbers is greater (see also Nuerk et al. 2015). Such ‘decade effects’ can also be observed in mental addition tasks, where the time and accuracy for performing additions of two-digit numerals can be explained by the structure of the numerals instead of the numerical values of the addends (Neth 2004). These empirical results show that accidental features of the decimal place-value notation, such as the fact that nine is represented by a single digit but ten is represented by two digits, are integrated in our cognitive processes (Schlimm 2018).

### Non-derived mathematical content

Not only are the symbolic vehicles of representation internalized over time, but they can also induce new content. For example, Menary (2015, p. 15) notes that once a public symbolic system for counting numbers is in place, this motivates the introduction of all sorts of ‘exotic’ numbers and operations that do not have their origin in previous mental content. The historical introduction of negative numbers, the number zero (as opposed to a placeholder symbol with no numerical content), complex numbers, etc., are all instances of this phenomenon. These external public systems allow for computations that could not be performed by any basic, biologically inherited number system (Gaber and Schlimm 2015). Recall how the concept of evolutionary continuity, central to the cognitive integration approach, applies to both cultural and biological evolutionary processes—this is key, as biology evolves at a pace too slow to explain the relatively rapid progress in mathematics. Menary (2015) argues that, for example, the neural circuits responsible for numerosity cannot represent either negative numbers or square roots. These concepts have to arise in conjunction with the public mathematical symbols that have been developed to represent them. This is because of the novelty and uniqueness of these symbolic representations. Novelty, Menary (2015, p. 15) explains, arises from external pressures, such as social and economic complexities.

A major driving force behind mathematical developments are, in addition to worldly problems for which a mathematical answer is sought, inner-theoretical pressures (Ferreirós 2015). These arise from various theoretical ideals, such as simplifying solution strategies, using as few assumptions as possible, avoiding unnecessary computations, and formulating general solutions that are not prone to exceptions. For example, any two counting numbers (i.e., positive integers), can be added to obtain another such number, just as any two collections of things can be combined to obtain a larger collection. Thus, addition of natural numbers can always be applied without any restrictions. However, this is not the case for subtraction. While 6 − 2 = 4, there is no natural number that expresses the result of subtracting 6 from 2. Analogously, there is no collection of oranges that represents the result of starting with 2 oranges and taking away 6 of them. In bookkeeping, this problem was solved by introducing the notions of debit and credit, both of which could be expressed with natural numbers alone. However, the desire to formulate a general theory that has as few exceptions as possible led mathematicians to the introduction of a symbol for expressing the result of subtracting 6 from 2, namely ‘−4’, and to formulate rules of operation for manipulating this new kind of symbol in conjunction with those symbols that express natural numbers, for instance, (−4) + 6 = 2. In a further step, these new symbols were not only thought of as being determined by formal rules, but also as referring to abstract entities, just like the ordinary numerals. In this way, *negative numbers* came into being, and, with them, almost any expression of the form *x*–*y* became meaningful, with *x*–*x* remaining the only exception. To fill this gap, also *zero* had to be considered to be a number and not just a placeholder symbol that signifies the absence of a value. Now, finally, mathematicians had developed an abstract domain of numbers, the integers, in which not only addition could be performed without exceptions, but also subtraction. This development was not driven primarily by social or economic pressures, but by inner-mathematical, theoretical ones (Corry 2015).

When manipulating algebraic equations, it is generally allowed to perform the same operation, such as addition, multiplication, and exponentiation, on both sides of an equation. For example, beginning with *x*^2^ + 1 = 0, subtracting 1 on both sides yields *x*^2^ = −1. But which number *x* can be multiplied with itself to result in negative one? Algebraists in the sixteenth century, such as Cardano and Bombelli, faced this question head on and came to the conclusion that expressions like *x *= √−1 should be accepted as solutions of algebraic equations, even though the symbolic expression of the square root of a negative number was considered ‘sophistic’ and ‘as subtle as it is useless’ (Corry 2015, p. 145). Nevertheless, such expressions proved their worth in the work of the algebraists and thus gained more and more acceptance. With time, rules for the operations with such expressions were codified, and the symbolic expressions were simplified by simply writing *i* for √−1. Later on, through the use of pairs of real numbers and operations on these pairs, the new expressions could be systematically related to better understood mathematical objects and they were granted the status of numbers—*imaginary* ones. In short, by relying on culturally established rules for manipulating mathematical expressions, mathematicians introduced symbolic expressions that were not imbued with some prior mental content. However, once such expressions proved their worth in a practice, for example by facilitating the solution of algebraic equations, new ideas were sought to fill them with content. Nowadays, *i* and √−1 have the same content, although this content was not fully understood by early users of these symbols, including the expert mathematicians who introduced them in the first place. The development of imaginary numbers is thus an example of how mathematicians sometimes introduce new symbols to represent new mathematical possibilities that are not yet understood. Krämer (2003) calls this process ‘operative writing’: what the notation represents—that is, the content of the symbol(s)—is constituted by the symbol itself and its interaction with other symbols. This is especially persuasive in cases where new symbols are introduced and manipulated within an established practice but in ways that were not anticipated before, such as subtracting a larger number from a smaller, taking the root of a negative number, or raising to the power where the exponent is a fraction or a real number (De Cruz and de Smedt 2013).

It is interesting to notice how the case of a mathematician who introduces a new symbol and who, together with a few other expert mathematicians in their small community, comes to develop an understanding of the content of the new symbol by manipulating it in accordance with their agreed-upon practice and transformation rules is parallel to the gang-slang case given above. Both are cases of *active content externalism*. The key difference from the gang-slang case is that these mathematical cases are not hypothetical but are recurring episodes in the history of mathematics. Such cases of operative writing, thus, challenge not only the objection from non-derived content but also Adams and Aizawa’s position of contingent intracranialism.

### Non-Euclidean geometries

The examples of the introduction of new mathematical domains discussed in the previous section were all motivated by the desire to apply arithmetical operations, such as subtraction and the taking of roots, without any of the restrictions imposed on them in the realm of whole numbers. We have argued that these extensions resulted from first introducing new symbols and then imbuing them with meanings and that the new mathematical content should be considered to be non-derived, because the external symbolic representations were prior to the mathematical conceptions that followed them. We now present a different case that is not based on domain extension, namely the introduction of non-Euclidean geometries.^18^

For over 2000 years after the publication of Euclid’s *Elements*, his axioms and theorems were considered to express geometric truths about the world. Most of the foundational research in geometry in the wake of Euclid was concerned with finding alternative axiomatizations with simpler and fewer axioms. In particular, his fifth postulate, also called ‘parallel postulate’ (Euclid distinguished between common notions and postulates, instead of talking about axioms), attracted a lot of attention from commentators from early on (Heath 1956, pp. 202–220). It states that ‘if a straight line falling on two straight lines make the interior angles on the same side less than two right angles, the two straight lines, if produced indefinitely, meet on that side on which are the angles less than the two right angles’ (as quoted in Heath 1956, p. 202). It already stands out from the remaining axioms of Euclid by the complexity of its formulation, and so mathematicians tried to simplify it or to prove it from the other axioms. This history is very well known, so we skip many details and simply sketch the main development. One way of showing that a statement follows from some given assumptions is by an indirect proof (proof by contradiction), that is, to assume the contrary of the statement together with the given assumptions and derive a contradiction. Accordingly, mathematicians, such as Saccheri, started by assuming the contrary of the parallel postulate, something that they wholeheartedly believed to be false. In other words, they thought that their assumptions taken together would be contradictory and thus they would have no content whatsoever. However, in the course of these investigations, all contradictions that were found were shown to be based on faulty reasoning; over time, the belief that the system of assumptions was indeed contradictory became weaker and weaker, and mathematicians such as Gauss, Lobachevsky, and Bolyai became convinced that this system was actually consistent after all. They talked about lines and planes for which the parallel postulate did not hold and deduced theorems about them. Later, Beltrami, Klein, and Poincaré all showed how these ‘non-Euclidean’ objects could be systematically related to the familiar Euclidean points, lines, and planes. By the end of the nineteenth century, non-Euclidean geometries were accepted as genuine mathematical domains (Gray 2008).

What the development of non-Euclidean geometries illustrates is a case where statements thought to express no mathematical content gradually became accepted vehicles of content. The fact that formal derivations from these assumptions did not lead to any contradictions suggested their contents. Unlike the examples in the previous section, non-Euclidean geometries do not extend Euclidean geometry but are in fact incompatible with it, which contributed to a radical change in mathematical ontology (Gray 1992). The linguistic community of mathematicians who developed the theories of non-Euclidean geometry in the nineteenth century was relatively small, and they were themselves the experts regarding the content of the axiom systems they were studying, again just like in the gang-slang case. Indeed, many philosophers were not familiar with the work of these experts; they continued to rely on their a priori conceptions of geometry and thus failed to grasp the content introduced by these novel developments. Thus, we may speak here of an example of active content externalism.

## Discussion of the notion of derived content

In introducing the distinction between derived and non-derived content, Adams and Aizawa (2001, p. 48) consider the case of numerals and describe them as having derived content: ‘Numerals of various sorts represent the numbers they do in virtue of social agreements and practices.’ Adams and Aizawa might, therefore, dismiss the examples we have given of numerals and postulates by arguing that they derive their content from convention or social practice. This brings out an important distinction between two senses of ‘derived’ content that Adams and Aizawa use. They say that non-cognitive representational states derive their meanings either (1) from representational states with intrinsic content—therefore genuinely cognitive representational states, or (2) from conventions or social practices. On the first definition, a symbol with derived content owes its meaning to an act of interpretation by an agent who has cognitive states bearing intrinsic content—states that do not need to be interpreted and that mean what they do independent of other representational states. On the second definition, the contents of non-cognitive representational states represent in virtue of convention or social practices, unlike non-derived content, which is supposed to be naturalistic and non-conventional (Piredda 2017). Adams and Aizawa do not clearly distinguish these two definitions of derived content—we have mentioned both of these definitions in our earlier introduction of derived content, but we have not carefully distinguished them until now.^19^ In the cases we described, the content of newly introduced symbols might come to be understood through the manipulations of the symbols in accordance with agreed-upon social practices, but their content cannot be derivative of any internal biologically instantiated representational states or processes, as the symbols are introduced originally without any clear internal understanding of their representational content, and their content is determined only by the syntactic manipulations that can be performed with them. Hence, we have shown that mathematical symbols are not derived in the first sense, and the objection we now consider is whether they are derived in the second sense, that is, derived from conventional associations or social practices. We will offer several responses to this objection.

First, if the distinction between derived and non-derived content is based only on the second definition, this seems to beg the question against the possibility of external symbols with non-derived content. Without an explanation as to how naturalistic content is possible, the claim that all and only biologically instantiated vehicles of representation have non-derived content, coupled with the claim that non-derived content is the mark of the cognitive, takes a position on the very question at hand—that is, where the vehicles of cognitive representations can be located. Even if there is consensus that neural representations do not depend on an act of interpretation to acquire meaning, the contingent intracranialists, without an explanation as to how this is the case, risk assuming what they are arguing for. They cannot simply assert a priori that all private representations, or internal representations, will have intrinsic content, and all representations external will have non-derived content (Prinz and Clark 2004; Clark 2005). As we have said, offering a naturalistic account of content continues to be a big issue in philosophy of cognitive science, although attempts have been made by Dretske (1981, 1988), Fodor (1987, 1990), Millikan (1984), and others.

Another way to push back on Adams and Aizawa’s assertion that all and only biologically instantiated vehicles of representation have non-derived content is to argue that at least some internal representations acquire their meaning through convention. We can imagine someone who relies on an internal ‘mental picture’ of words, such as ‘dog’, in her thinking about dogs. This linguistic representation derives its meaning by convention, and, yet, it occurs internally. Clark (2005, p. 5) gives the example of ‘an episode of in-the-head problem solving during which [one] imagine[s] the partially overlapping circles of a certain Venn diagram. Surely the set-theoretic meaning of this overlap is a matter of convention? Yet the images figure centrally in what is surely a cognitive process in good standing.’ Also, the empirical results discussed in Sect. 8.2, where the performance in mental arithmetic tasks clearly depends on the structure of the external decimal-place value notation of numerals, illustrate this point. In all of these cases, biologically instantiated representational vehicles have content that is derived by convention, yet we call these relevant information-bearing structures cognitive because they are located in the brain—this neuro-chauvinistic attitude is motivating the contingent intracranialist position.

Finally, we suggest that a further distinction needs to be made between two ways in which a symbol can have content through convention: (a) by being assigned some previously given content, and (b) where the content itself emerges from social conventions about the use of the symbol. In the first case, an arbitrary symbol is assigned a given content and a convention is established to use that symbol to represent that content. An example is the linguistic representation for dog, namely, the word ‘dog’. Furthermore, a social practice is established within a particular community—English speakers—to use ‘dog’ to refer to a dog. This case is importantly different from the mathematical cases we have described, which are instances of symbols that emerge from social conventions. In the cases of −4 and *i*, the symbols were introduced before the content of the symbols was determined. In other words, there was no prior content that was assigned to the symbols −4 and *i*; rather this content had to emerge from the symbolic practice of manipulating the symbols through the process of operative writing and by linking them to other mathematical domains. Thus, in these cases, the content itself stems from the social practices, an aspect of the process of enculturation, that are external to any individual thinker and that guide the manner of manipulating mathematical symbols. Likewise, non-Euclidean geometries emerged from the explicit rejection of an established convention—Euclid’s parallel postulate—and emerged through the cognitive practices of mathematicians, who themselves believed the contrary of the parallel postulate to be false, or void of mathematical content. Reluctantly, through mathematical manipulations, they came to see that they were mistaken. These cases are importantly different from how other external symbols acquire content through convention and, we argue, they provide a case in which the content of external symbols precedes any conventional association or any act of interpretation by a cognitive agent.

## Conclusion

Menary’s theory of cognitive integration is a version of vehicle externalism and, as such, it faces a longstanding objection to vehicle externalism as raised by Adams and Aizawa. Their objection from non-derived content claims (a) that non-derived content is a mark of the cognitive and (b) that, as a matter of contingent fact, no external vehicles of representations can have non-derived content. We have argued against the second claim, demonstrating instead that there are cases in mathematics where external symbols have content that is not derived either from conventional associations or from the representational states of a cognitive agent.

While Menary considers mathematical cognition as a primary example of the process of enculturation, which is a central aspect of his cognitive integrationist framework, he does not develop this view as a response to the objection from non-derived content. This has been our aim in this paper. Our response to the objection relied on two sorts of historical cases: the first demonstrates how expert mathematicians introduce new symbols to represent mathematical possibilities that are not yet understood; the second case moves away from what could be considered mere domain extensions and instead shows the development of an entirely new domain, non-Euclidean geometry, that was premised on the rejection of previously established mathematical assumptions. These cases are examples of what we call active content externalism—they show how a symbol can drive understanding, even when the content of the symbol is not yet understood by those who introduce it. If correct our argument would establish that there are at least some cases of external representations with non-derived content and, hence, that vehicle externalism is possible (though it would not be a defense of all of the popular examples of vehicles externalism, such as the case Otto’s notebook).

We ended by considering a possible objection, namely, that our examples of external symbols have content that is derived by convention and social practices, rather than being derived in the sense of requiring an act of interpretation. In response to this we argued, first, that this sense of ‘derived’ may beg the question and, second, that there are in fact two senses in which content can be determined by convention—one in which an understood content is somewhat arbitrarily assigned to a symbol and subsequently a convention is established; and another in which a new symbol is introduced without a known content and social practices then come to determine that content. The second sense of ‘derived’, we argue, is far more interesting and should be used for genuine cases of non-derived content; we illustrated such cases in our examples of mathematical symbols and axioms. If we are right, then we have grounds to reject Adams and Aizawa’s position of contingent intracranialism. As a consequence, the onus would now fall on the embedded mind theorist, who supports intracranialism, to find a principled reason for rejecting vehicle externalist views, such as the extended mind thesis and the theory of cognitive integration.

## Footnotes

1 Other versions of vehicle externalism have been defended by philosophers such as Chemero (2009), Clark (1996), Hurley (1998a, b, 2010), Hutchins (1995), Noë (2004), Palermos (2014), Rowlands (2010), Wheeler (2005), and Wilson (1994, 2004).

2 Some maintain that cognitive states and processes are a subset of mental states and processes, while others insist that the two sets overlap. We do not take a position on this issue but use the terms ‘cognitive’ and ‘mental’ as interchangeable.

3 Sterelny (2003) describes the cognitive niche as the structured environment that humans have created (both the immediate niche that we create for ourselves and that which is created by others in the past and present) and that enhances our cognition in ways that are often transparent to us—as culture often is.

4 Menary (2015) focuses on four different aspects of enculturation—evolutionary continuity, transformation, learning-driven neural plasticity, and novelty or uniqueness. The first two play a role in our argument addressing Adams and Aizawa's objection to vehicle externalism, while the latter two do not. But because the concepts of neural plasticity and novelty or uniqueness are also central to enculturation, we will briefly touch on them here. First, neural plasticity highlights the role of developmental plasticity in the human brain, through Hebbian synaptic plasticity, growth of grey matter, and subsequent pruning during critical periods. What is key for our purposes is that developmentally plastic human brains need cognitively scaffolded environments to learn complex cognitive practices and techniques, including but not limited to, mathematical practices. Menary's concept of novelty, or uniqueness, emphasizes how external symbols and tools do not merely replace, or substitute for, our internal cognitive capacities but can also give as new, novel cognitive capacities. This could be said of basic tools such as an axe, which allows us to chop things our hands alone could not chop. But this could also be said of other basic technologies such as writing utensils, which allow us to create finely grained external symbols that allow us to publicly represent equally finely grained concepts. The development of mathematical cognition relies on all four features of enculturation: evolutionary continuity, behavioural and neural plasticity, transformation, and novelty or uniqueness.

5 Notably, Menary (2015, p. 6) is more cautionary about evolutionary claims that draw links between human brains and lower primates.

6 The cognitive integration framework, thus, does not focus on the brain as the centre of a cognitive system. This point helps to distinguish the scope of Menary’s view from that of Clark and Chalmers’s. According to Menary (2015, p. 12), brains are unique insofar as they make their own distinctive contributions to our cognitive life, but understanding human cognition requires looking at the ‘hybrid and multi-layered system’ that brings it about. This includes the distinctive processing of the brain, body, environment, and evolution. Comparatively, the extended mind thesis is rather brain-centred, as it takes the brain to be at the centre of the extended cognitive system. According to Clark (2003, 2011), brains are special because of (a) the central role they play in bringing about our mental lives and (b) their ability to integrate with non-biological props and aids. Thus, although it is compatible with the cognitive integration approach, Clark and Chalmers’s parity argument does not pay the same attention to evolutionary processes or cultural practices.

7 This point further distinguishes the cognitive integration version of vehicle externalism from that of Clark’s position, which does not maintain that our basic cognitive resources are transformed. Clark instead thinks that our biological resources ‘simply dovetail’ to public symbols (Menary 2015, p. 9). Arguably, this makes it difficult for Clark’s account to explain the learning process for cognitive tasks that require the manipulation of external symbols, such as mathematical cognitive tasks.

8 The view that Menary (2015, p. 2) calls the ‘moderate embedded mind thesis’ is what we refer to simply as the ‘embedded mind thesis’, while what Menary refers to as the ‘embedded mind strong’ we call vehicle externalism.

9 Advocates of the embedded view include, for example, Sterelny (2003, 2010; although he allows that there may be limited cases of genuine cognition extension) and Adams and Aizawa (2008), who accept the embedded claim but argue against cognitive extension.

10 For more on the debate between these two positions, see Adams and Aizawa (2001, 2008), Shapiro (2010), Sterelny (2010), and Clark (2008, 2010).

11 Adams and Aizawa (2001, pp. 48–53) defend non-derived content as a necessary condition for a state or process to be cognitive, along with the condition that cognitive processes are causally individuated. The objection we are concerned with in this paper stems from the first necessary condition only. This objection maintains that external states and processes do not have non-derived content and therefore are not cognitive.

12 In fact, Adams and Aizawa (2001, p. 49) are careful to distinguish the condition that cognitive processes must involve non-derived content from the thesis of representationalism. Thus, a stronger condition for the mark of the cognitive would be a conjunction of non-derived content and representationalism, which would maintain that cognition necessarily involves *representations* that have non-derived content. Adams and Aizawa believe in this stronger claim as well, but do not avail themselves of it. We will discuss this stronger claim as the mark of the cognitive because vehicle externalists also tend to endorse representationalism, as their position maintains that the vehicles of mental representations can be external to the biological organism.

13 These two senses of ‘derived’ in fact need to be pulled apart, a point which we will discuss towards the end of the paper.

14 Adams and Aizawa's claim is inspired by Searle’s (1980) more familiar distinction between intrinsic and derived intentionality. Although Clark (2005) points out one main difference: for Searle intrinsic intentionality applies to whole systems, while for Adams and Aizawa intrinsic content seems to be a feature of the parts (namely the states or processes) of cognitive systems rather than to the whole. Clark further argues that the concept of ‘intrinsic content’ is both unclear and a confused adaptation of Searle’s notion of intrinsic intentionality. Our argument in this paper will charitably accept the notion of ‘intrinsic content’ as a necessary condition for cognition but argue that, even so, it still does not give grounds for rejecting vehicle externalism.

15 Another version of this objection can be found in Fodor (2009), and a further discussion of the need for a mark of the mental can be found in Rowlands (2009), who includes original content as a necessary condition for cognition, along with three other necessary conditions.

16 Lyre (2016, pp. 24, 25) calls this position ‘extended internalism’ because it still accepts the supervenience claim that content *internalists* accept. Only the vehicles can be extended, so they might not be brain states or processes and may instead be external to the skin-and-skull, but the content still supervenes on the representational vehicles of (or within) the ‘extended’ cognitive system.

17 Lyre calls this ‘active social externalism’, but to reduce the number of terms we introduce, we will simply call this ‘active content externalism’. Active social externalism combines the social elements of Burge’s view with the active elements of Clark and Chalmers’s vehicle externalism.

18 This is an example of the creative use of axioms by modification (Schlimm 2011, 2013).

19 Neither does Clark (2005) in his reply to Adams and Aizawa, nor do secondary commentators, such as Piredda (2017), make the distinction between the two kinds of derived content.
